# Comparative population pharmacokinetics and absolute oral bioavailability of COX-2 selective inhibitors celecoxib, mavacoxib and meloxicam in cockatiels (*Nymphicus hollandicus*)

**DOI:** 10.1038/s41598-017-12159-z

**Published:** 2017-09-25

**Authors:** Laura Dhondt, Mathias Devreese, Siska Croubels, Siegrid De Baere, Roel Haesendonck, Tess Goessens, Ronette Gehring, Patrick De Backer, Gunther Antonissen

**Affiliations:** 1 Department of Pharmacology, Toxicology and Biochemistry, Faculty of Veterinary Medicine, Ghent University, Merelbeke, Belgium; 2 Department of Anatomy and Physiology, Institute of Computational Comparative Medicine, College of Veterinary Medicine, Kansas State University, Manhattan, KS USA; 3 Department of Pathology, Bacteriology and Avian Diseases, Faculty of Veterinary Medicine, Ghent University, Merelbeke, Belgium

## Abstract

Selective COX-2 inhibitors are non-steroidal anti-inflammatory drugs which directly target cyclooxygenase-2 (COX-2), an enzyme mainly responsible for induction of inflammation, pyresis and pain. Although commonly used in avian medicine, limited pharmacokinetic (PK) data in domestic and companion birds are available. In this study, PK parameters and absolute oral bioavailability expressed as percentage (F%) of celecoxib (10 mg/kg BW), mavacoxib (4 mg/kg BW) and meloxicam (1 mg/kg BW) were determined following single oral (PO) and intravenous (IV) administration to cockatiels *(Nymphicus hollandicus)*. The drugs were quantified in plasma by liquid chromatography-tandem mass spectrometry. Data were processed using the nonlinear mixed effects (NLME) approach. In contrast to celecoxib (T_1/2el_ = 0.88 h) and meloxicam (T_1/2el_ = 0.90 h), mavacoxib has a prolonged elimination half-life (T_1/2el_ = 135 h) following oral administration of a commercial formulation (CF). High to complete oral absorption was observed following oral administration of celecoxib (F% = 56–110%) and mavacoxib (F% = 111–113%), CF and standard solutions, respectively. In contrast, the F% of meloxicam was low (F% = 11%). Based on the presented results, a less frequent dosing of mavacoxib is proposed compared to celecoxib and meloxicam. However, pharmacodynamic and safety studies are necessary to further investigate the use of these NSAIDs in cockatiels.

## Introduction

Nonsteroidal anti-inflammatory drugs (NSAIDs) are commonly used in both veterinary and human medicine for their anti-inflammatory, analgesic and antipyretic properties. NSAIDs prevent conversion of arachidonic acid into prostanoids, including prostaglandins, prostacyclins and thromboxanes, by inhibiting the cyclooxygenase (COX) iso-enzymes. Two related isoforms of the COX enzyme have been described: cyclooxygenase-1 (COX-1) and cyclooxygenase-2 (COX-2). COX-1 is constitutively expressed in several tissues and is involved in the regulation of homeostatic functions throughout the body. In contrast, the expression of COX-2 is generally considered to be induced by inflammatory stimuli under different pathological conditions. However, in certain tissues like the kidney, brain and thymus, COX-2 is also constitutively expressed^1–3^. COX-1 and COX-2 are both targets of non-selective NSAIDs, whereas COX-2 selective NSAIDs inhibit selectively COX-2. These selective COX-2 inhibitors cause less side effects by preserving the COX-1 regulated homeostatic functions, including gastric cytoprotection, renal blood flow maintenance and platelet function^3–5^.

Avian pain management is characterized by multiple challenges. Recognizing pain and assessing its intensity are both essential for effective management. However, behaviour associated with painful stimuli is often subtle and not very specific in birds. Consequently, failure to appreciate the intensity of pain will confine the selection of an appropriate potent analgesic drug and corresponding dosing regimen^6^. Therefore, both owners and clinicians must be familiar with the normal behaviour of both the animal species and the individual bird to recognize signs of pain^7,8^. Secondly, pharmacokinetic (PK) processes of drugs, namely absorption, distribution, metabolism and excretion (ADME), differ largely between mammals and birds, and they also vary among avian species. Significant species differences in the primary PK properties of some NSAIDs, such as salicylic acid, flunixin, and meloxicam, show that PK data and also posology, can hardly be extrapolated from mammals to birds and among different bird species^8–10^. Third, the safety profile of NSAIDs may vary considerably among animal species, including birds^11,12^.

**Figure 1:**
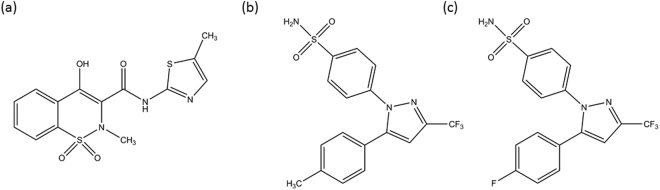
Molecular structure of meloxicam (**a**) celecoxib (**b**) and mavacoxib (**c**).

Notwithstanding these considerations, NSAIDs are commonly used in avian clinical practice to reduce pain and inflammation of various origins, including musculoskeletal, visceral and postoperative pain. Various NSAIDs, like meloxicam, piroxicam, carprofen, ketoprofen and celecoxib, have been used in birds to treat pain and inflammation, with meloxicam (Fig. 1a) as preferential COX-2 inhibitor being the most widely used in avian practice^8,13,14^. However, the usage is off-label. Meloxicam has been used in a dose range of 0.1–2.0 mg/kg body weight (BW) for various painful and/or inflammatory conditions, like arthritis, postoperative pain and proventricular dilatation disease (PDD)^15^. PDD is a progressive, infectious neurologic disease, mainly affecting psittacine birds. It is characterized by an avain bornavirus-induced inflammation of the digestive tract as well as the central and peripheral nervous system. This inflammation leads to a variability of clinical signs such as weight loss, crop stasis, regurgitation, maldigestion, and eventually starvation and death^16,17^. Since inflammation is the key pathologic event, NSAIDs may have a therapeutic value in the symptomatic treatment of PDD. PK studies conducted in several avian species showed a wide interspecies variability in PK properties of meloxicam. Significant PK variations between chickens *(Galllus gallus)*, ostriches *(Struthio camelus)*, ducks *(Anas platyrhynchos)*, turkeys *(Meleagris gallopavo)*, pigeons *(Columba livia)*, Hispaniolan Amazon parrots *(Amazona ventralis)* and African grey parrots *(Psittacus erithacus)* have been reported. Divergent values of clearance (Cl) and volume of distribution (Vd) were observed in these species, ranging from 2.18 to 720 mL/h.kg and 58 to 580 mL/kg respectively^10,15,18^. Furthermore, allometric scaling showed poor correlations between BW and these PK parameters in different avian species^10^.

The coxibs, such as celecoxib and mavacoxib, have a highly specific action on the COX-2 enzyme. In humans, celecoxib (Fig. 1b) is indicated in case of osteoarthritis, rheumatoid arthritis, ankylosing spondylitis and acute pain. Its use in veterinary medicine is off-label, however, celecoxib in a dose of 10 mg/kg BW is used in clinical practice for the symptomatic treatment of PDD in birds. Although no peer-reviewed studies about the use of celecoxib in case of PDD are available, it is considered as the standard therapy. Mavacoxib (Fig. 1c), registered for use in dogs with osteoarthritis, is characterized by a metabolically stable fluoro substituent at the position were its structural analogue celecoxib has a metabolically labile aromatic methyl group. Consequently, in dogs a low clearance and prolonged elimination half-life (t_1/2el_) are reported. Therefore a less frequent dosing is necessary, which contributes to an improved therapeutic compliance^19–21^. Initially, a classical dose determination study indicated that a dose of 4 mg/kg BW is necessary to exhibit clinical efficacy. More recently, population PK (popPK) data showed that reducing the dose from 4 mg/kg BW to 2 mg/kg BW in combination with a non-constant dosing interval (2 weeks between the first two doses; thereafter maintenance interval of 4 weeks) was sufficient to attain and maintain therapeutic concentrations^19,22^. To date, no information on the use of mavacoxib in avian species has been described in literature.

The purpose of this study was first to develop and validate a sensitive and specific liquid chromatography-tandem mass spectrometric (LC-MS/MS) method for quantification of meloxicam, celecoxib and mavacoxib in avian plasma. Second, the PK properties and absolute oral bioavailability of celecoxib, mavacoxib and meloxicam in cockatiels *(Nymphicus hollandicus)* after single intravenous (IV) and oral (PO) administration using a popPK approach were assessed.

## Results

### LC-MS/MS methods

Linear matrix-matched calibration curves, covering a concentration range of 5–5000 ng/mL (celecoxib and mavacoxib) and 10–5000 ng/mL (meloxicam), were obtained. Good correlation between analyte concentrations and detected responses was demonstrated for all compounds, with correlation coefficient (r) values ranging between 0.9982 and 0.9999 and goodness-of-fit coefficient (gof) values between 4.95 and 6.25% (see Supplementary Table S1). The acceptance criteria for within- and between-run accuracy and precision were met for all drugs at the specified concentration levels (see Supplementary Table S2). The limits of quantification (LOQ) were 5 ng/mL for celecoxib and mavacoxib, and 10 ng/mL for meloxicam. The calculated limit of detection (LOD) values, corresponding with a signal/noise (S/N) ratio of 3, were 0.22, 0.25 and 0.18 ng/mL for celecoxib, mavacoxib and meloxicam, respectively (see Supplementary Table S1). Since no interfering peaks could be detected in any of the blank samples at the retention time of the drugs, the specificity of the method was demonstrated. No carry-over was present as there was no analyte detected in the solvent sample injected after the highest calibrator.

### Pharmacokinetic analysis

**Figure 2:**
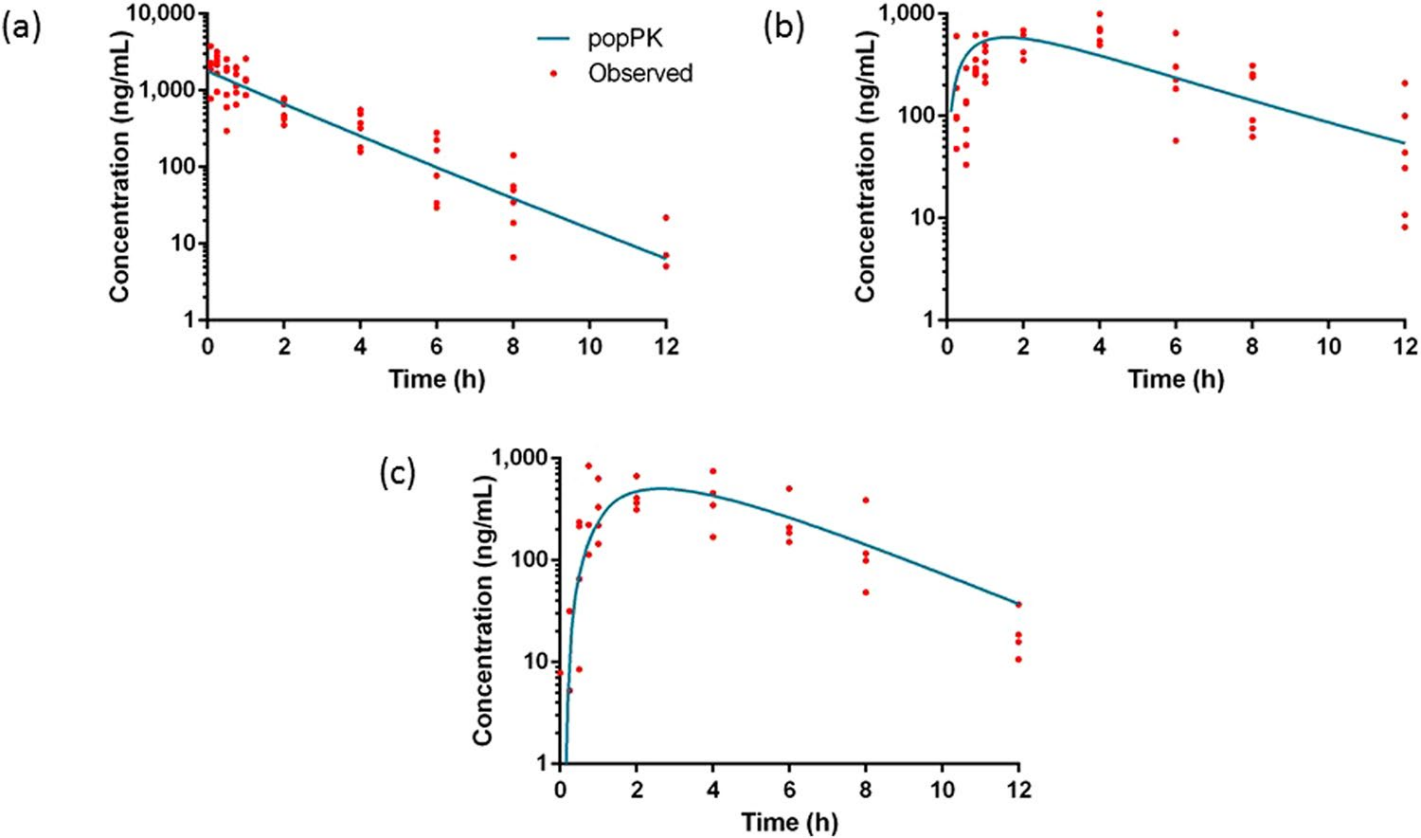
Pharmacokinetic profiles of celecoxib. Observed and modeled plasma concentration-time profiles of celecoxib (10 mg/kg BW) after IV (n = 34) (**a**) and PO (n = 34) (**b**) administration of the standard solution and of the PO administration of the commercial formulation (n = 22) (**c**) in cockatiels.

**Figure 3:**
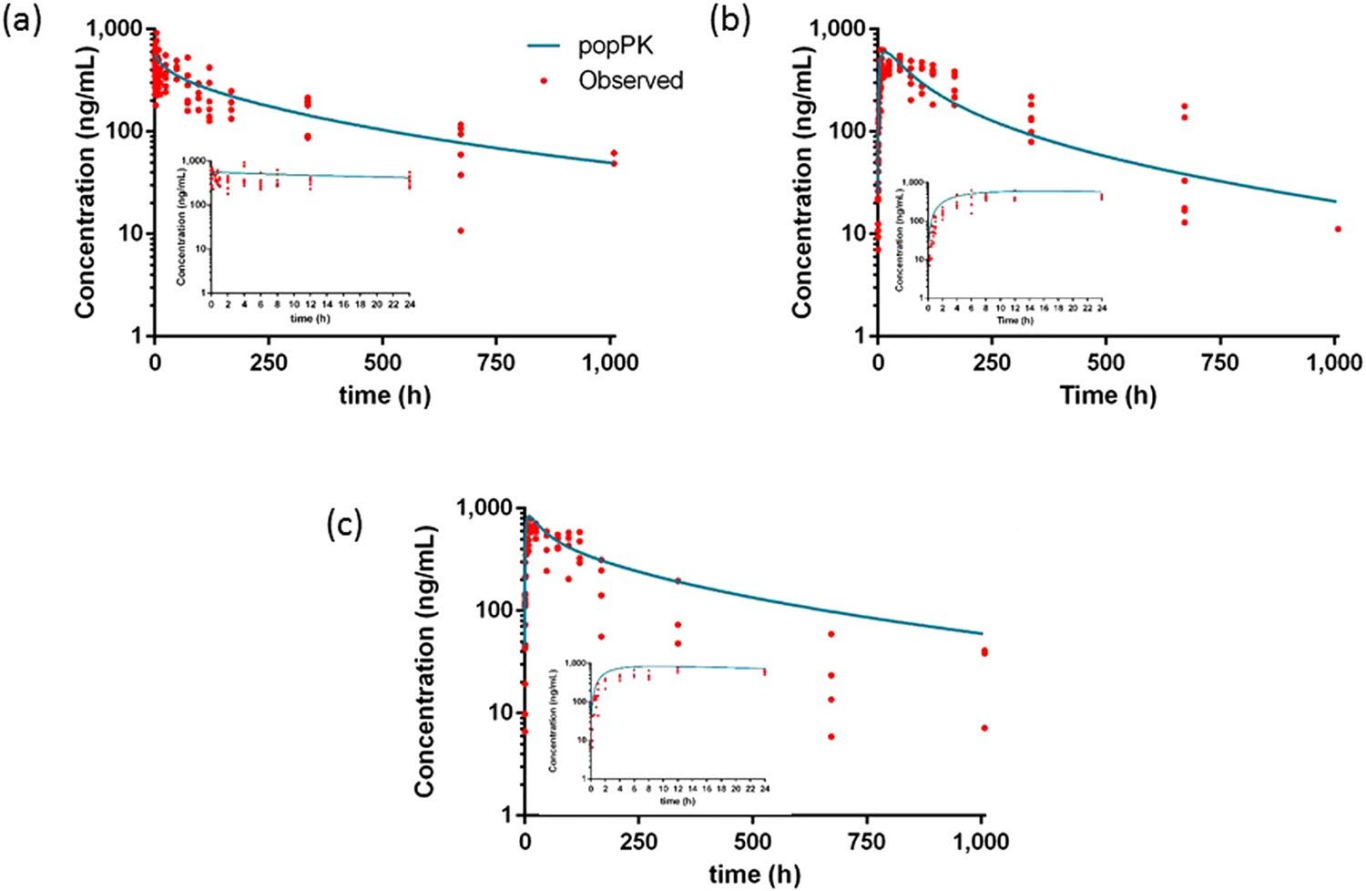
Pharmacokinetic profiles of mavacoxib. Observed and modeled plasma concentration-time profiles of mavacoxib (4 mg/kg BW) after IV (n = 40) (**a**) and PO (n = 40) (**b**) administration of the standard solution and of the PO administration of the commercial formulation (n = 26) (**c**) in cockatiels. The insert shows the first 24 h of the plasma-concentration-time profiles.

**Figure 4:**
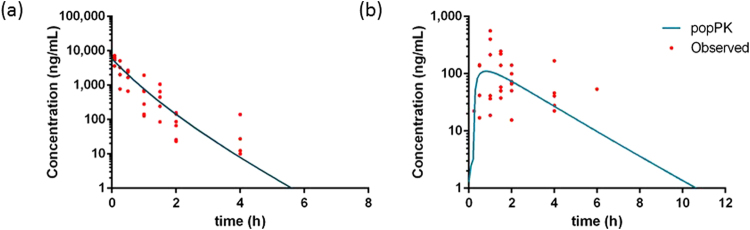
Pharmacokinetic profiles of meloxicam. Observed data and modeled plasma concentration-time profiles of meloxicam (1 mg/kg BW) after IV (n = 24) (**a**) and PO (n = 24) (**b**) administration of the commercial formulation in cockatiels.

During the animal experiment no clinical signs of toxicity were observed in any of the birds. All birds were alert, had a normal feed intake and normal droppings, and no regurgitation was observed. Plasma drug concentration-time profiles of celecoxib, mavacoxib and meloxicam are shown in Figs 2, 3 and 4, respectively. PopPK results are presented in Tables 1, 2 and 3. For the oral CF of celecoxib and meloxicam, a lag-time was included since this significantly improved the model fit. The evaluated covariates, BW and gender, were not significant for addition to the PK models for any of the drugs. Inclusion of enterohepatic recirculation (EHR) in the model of meloxicam did not significantly improve the model fit (−2 log likelihood (−2LL), Akaike’s information criterion (AIC) and Bayesian information criterion (BIC)) (Table 4). Although EHR has been demonstrated for several animal species, this lack of improvement is most probably attributed to the limited blood collection points at the secondary plasma concentration peak. Visual inspection of the goodness-of-fit plots of the individual model-predicted concentrations (IPRED) versus the observed concentrations (C_obs_) revealed an appropriate structural model for most individuals. The quantile-quantile (QQ) plots of the conditionally weighted residuals of C_obs_ demonstrated normal distribution of the weighted residuals (see Supplementary Fig. S1–S3).

**Table 1:** Population pharmacokinetic results of intravenous (IV) and oral (PO) administration of celecoxib (10 mg/kg BW) to cockatiels using a sparse sampling protocol.

Adm. Route	Θ	Tvθ	CV (%)	ω
IV _STD_	Vd (L/kg)	4.67	8.06	<0.001
	Cl (L/h.kg)	2.42	7.93	0.0418
	C_0_ (ng/mL)	2141.54	8.06	/
	AUC_0-inf_ (ng.h/mL)	4140.36	7.93	/
	K_e_ (h^−1^)	0.52	5.40	/
	T_1/2el_ (h)	1.34	5.40	/
	Res. Error	0.42	12.32	/
PO _STD_	Vd/F (L/kg)	5.87	17.43	0.948
	Cl/F (L/h.kg)	2.19	17.86	0.707
	K_a_ (h^−1^)	0.27	8.98	0.063
	T_1/2a_ (h)	2.51	8.98	/
	C_max_ (ng/mL)	535.51	10.95	/
	T_max_ (h)	3.11	14.09	/
	AUC_0-inf_ (ng.h/mL)	4573.68	17.56	/
	K_e_ (h^−1^)	0.37	24.38	/
	T_1/2el_ (h)	1.86	1.86	/
	Res. Error	8.02	34.93	/
PO _CF_	Vd/F (L/kg)	5.49	25.02	<0.001
	Cl/F (L/h.kg)	4.32	11.67	0.055
	T_lag_ (h)	0.33	39.55	0.207
	K_a_ (h^−1^)	0.39	17.37	<0.001
	σ_mult_	0.41	18.17	/
	T_1/2a_ (h)	1.76	17.37	/
	C_max_ (ng/mL)	454.97	14.27	/
	T_max_ (h)	2.09	8.97	/
	AUC_0-inf_ (ng.h/mL)	2312.24	11.67	/
	K_e_ (h^−1^)	0.79	29.30	/
	T_1/2el_ (h)	0.88	29.31	/
	Res. Error	15.18	35.72	/

STD: analytical standard; CF: commercial formulation; Θ: fixed effect parameter; tvθ: population typical value of the fixed effect parameter; CV%: coefficient of variation; ω: variance of the interindividual variability (only for fixed parameters); Vd: volume of distribution; Cl: total body clearance; C_0_: plasma concentration at time 0 following IV administration; AUC_0-inf_: area under the plasma concentration-time curve from time 0 to infinity; K_e_: elimination rate constant; T_1/2el_: elimination half-life; Res. Error: residual error; Vd/F: volume of distribution uncorrected for absolute oral bioavailability; Cl/F: total body clearance uncorrected for absolute oral bioavailability; K_a_: absorption rate constant; T_1/2a_: absorption half-life; C_max_: maximal plasma concentration; T_max_: time to maximal plasma concentration; T_lag_: lag time for absorption following oral administration; σ_mult_: variance of the multiplicative residual error.

**Table 2:** Population pharmacokinetic results of intravenous (IV) and oral (PO) administration of mavacoxib (4 mg/kg BW) to cockatiels using a sparse sampling protocol.

Adm. Route	Θ	Tvθ	CV (%)	ω
IV _STD_	Vd (L/kg)	10.99	5.51	0.075
	Cl (L/h.kg)	0.036	12.16	0.524
	C_0_ (ng/mL)	363.75	5.51	/
	AUC_0-inf_ (ng.h/mL)	111238.0	12.16	/
	K_e_ (h^−1^)	0.0033	13.55	/
	T_1/2el_ (h)	211.97	13.55	/
	Res. Error	0.26	13.74	/
PO _STD_	Vd/F (L/kg)	7.85	4.26	0.006
	Cl/F (L/h.kg)	0.031	10.80	0.260
	K_a_ (h^−1^)	0.20	14.80	0.171
	T_1/2a_ (h)	3.48	14.80	/
	C_max_ (ng/mL)	469.69	3.35	/
	T_max_ (h)	19.98	10.19	/
	AUC_0-inf_ (ng.h/mL)	126108.0	10.80	/
	K_e_ (h^−1^)	0.0040	12.44	/
	T_1/2el_ (h)	171.68	12.44	/
	Res. Error	0.29	14.40	/
PO _CF_	Vd/F (L/kg)	6.35	0.19	0.090
	Cl/F (L/h.kg)	0.033	1.35	0.252
	K_a_ (h^−1^)	0.28	9.97	0.992
	T_1/2a_ (h)	2.44	9.97	/
	C_max_ (ng/mL)	584.66	0.32	/
	T_max_ (h)	14.42	7.94	/
	AUC_0-inf_ (ng.h/mL)	122962.0	1.35	/
	K_e_ (h^−1^)	0.0051	1.16	/
	T_1/2el_ (h)	135.41	1.17	/
	Res. Error	0.28	39.64	/

STD: analytical standard; CF: commercial formulation; Θ: fixed effect parameter; tvθ: population typical value of the fixed effect parameter; CV%: coefficient of variation; ω: variance of the interindividual variability (only for fixed parameters); Vd: volume of distribution; Cl: total body clearance; C_0_: plasma concentration at time 0 following IV administration; AUC_0-inf_: area under the plasma concentration-time curve from time 0 to infinity; K_e_: elimination rate constant; T_1/2el_: elimination half-life; Res. Error: residual error; Vd/F: volume of distribution uncorrected for absolute oral bioavailability; Cl/F: total body clearance uncorrected for absolute oral bioavailability; K_a_: absorption rate constant; T_1/2a_: absorption half-life; C_max_: maximal plasma concentration; T_max_: time to maximal plasma concentration.

**Table 3:** Population pharmacokinetic results of intravenous (IV) and oral (PO) administration of meloxicam (1 mg/kg BW) to cockatiels using a sparse sampling protocol.

Adm. Route	Θ	Tvθ	CV (%)	ω
IV _CF_^1^	Vd (L/kg)	0.173	11.74	<0.001
	Cl (L/h.kg)	0.388	10.19	0.089
	C_0_ (ng/mL)	5775.52	11.75	/
	AUC_0-inf_ (ng.h/mL)	2575.66	10.19	/
	K_e_ (h^−1^)	2.24	8.74	/
	T_1/2el_ (h)	0.31	8.74	/
	Res. Error	0.39	26.43	/
IV _CF_^2^	Vd (L/kg)	0.148	10.15	<0.001
	Cl (L/h.kg)	0.383	9.81	0.113
	K_a_ (h^−1^)	7.35	18.23	0.020
	K_1g_ (h^−1^)	0.40	31.43	1.153
	τ (h)	0.62	9.30	<0.001
	C_0_ (ng/mL)	385.515	10.15	/
	AUC_0-inf_ (ng.h/mL)	149.05	9.81	/
	Res. Error	0.36	22.79	/
PO _CF_^1^	Vd/F (L/kg)	4.40	27.97	<0.001
	Cl/F (L/h.kg)	3.38	18.82	0.122
	T_lag_ (h)	0.23	8.60	<0.001
	K_a_ (h^−1^)	1.19	55.88	1.075
	T_1/2a_ (h)	0.58	55.88	/
	C_max_ (ng/mL)	102.31	19.12	/
	T_max_ (h)	1.27	17.32	/
	AUC_0-inf_ (ng.h/mL)	295.26	18.81	/
	K_e_ (h^−1^)	0.77	32.34	/
	T_1/2el_ (h)	0.90	32.34	/
	Res. Error	1.15	14.19	/

CF: commercial formulation, ^1^no enterohepatic recycling included in the model; ²with enterohepatic recycling inclusion in the model; Θ: fixed effect parameter; tvθ: population typical value of the fixed effect parameter; CV%: coefficient of variation; ω: variance of the interindividual variability (only for fixed parameters); Vd: volume of distribution; Cl: total body clearance; K_a_: absorption rate constant; K_1g_: bile excretion rate; τ: bile excretion interval; C_0_: plasma concentration at time 0 following IV administration; AUC_0-inf_: area under the plasma concentration-time curve from time 0 to infinity; K_e_: elimination rate constant; T_1/2el_: elimination half-life; Res. Error: residual error; Vd/F: volume of distribution uncorrected for absolute oral bioavailability; Cl/F: total body clearance uncorrected for absolute oral bioavailability; T_1/2a_: absorption half-life; C_max_: maximal plasma concentration; T_max_: time to maximal plasma concentration; T_lag_: lag time for absorption following oral administration.

**Table 4:** Comparison of the −2 log likelihood criterion (−2LL), Akaike’s (AIC) and Bayesian information criterion (BIC) of the final population models of intravenous meloxicam whether or not including enterohepatic recycling (EHR) in the model.

	−2LL	AIC	BIC
no EHR	521.72	531.72	539.36
including EHR	518.14	542.13	560.45

The absolute oral bioavailability expressed as percentage (F%) of celecoxib was 110% and 56%, standard solution (STD) and commercial formulation (CF), respectively. For mavacoxib, F% was 113% (STD) and 111% (CF). The F% of meloxicam, when EHR was not taken into account, was 11%. Vd and Cl after oral administration were corrected for the absolute oral bioavailability and were in most cases similar between the different routes of administration and formulations. Because of that similarity and to reduce the complexity of the paper, mainly IV data is further provided. Important differences in PK parameters were observed when comparing the three NSAIDs following IV administration. In contrast to meloxicam (0.173 L/kg), the observed Vd of the coxibs, celecoxib (4.67 L/kg) and mavacoxib (10.99 L/kg), were high. Although the strong structural relationship, mavacoxib had a larger Vd than celecoxib. Due to its metabolic resistance, mavacoxib (0.036 L/h.kg) possesses a remarkably low Cl. The observed Cl of celecoxib (2.42 L/h.kg) is higher than meloxicam (0.388 L/h.kg). As a result of the combination of a high Vd and low Cl the T_1/2el_ of mavacoxib (212 h) exceeded to a considerable degree the T_1/2el_ of celecoxib (1.34 h) and meloxicam (0.31 h).

Plasma protein binding (mean ± SD) was high, namely 98.98 ± 0.07%, 97.02 ± 0.32% and 95.02 ± 0.01% for celecoxib, mavacoxib and meloxicam, respectively.

## Discussion

For the first time, a comparative study was performed to evaluate the PK of three different COX 2 inhibitors, celecoxib, mavacoxib and meloxicam, in cockatiels *(Nymphicus hollandicus)* as a model for the order of Psittaciformes. Besides, cockatiels are frequently used as model in PDD studies. The doses of celecoxib (10 mg/kg BW), mavacoxib (4 mg/kg BW) and meloxicam (1 mg/kg BW) used in the present study were based on previous literature reports^15,19^. Since a large number of samples need to be analysed in PK studies, high-throughput sample preparation is crucial. The application of the Ostro^TM^ 96-well plate for meloxicam and liquid-liquid extraction for celecoxib and mavacoxib is straightforward and relatively quick to perform. The combination of these robust sample preparation methods with LC-MS/MS allows a sensitive and selective quantification of celecoxib, mavacoxib and meloxicam in avian plasma. The presented results of the different PK parameters of the selected NSAIDs are opening perspectives for clinical use in birds, although further specific pharmacodynamic and safety studies are necessary. Given the limited blood volume in cockatiels, a sparse sampling protocol was applied which renders popPK modelling using non-linear mixed-effects (NLME) analysis necessary. The application of popPK modelling offers the possibility to collect representative samples while minimizing distress and blood loss, which is of pivotal importance in small and exotic bird species^23^. Complete drug absorption was demonstrated following oral administration of mavacoxib STD and CF (F% = 113 and 111%, respectively). In contrast, the F% of celecoxib after CF administration (56%) was notably lower than after STD administration (110%). A remarkable finding was the considerable low F% of meloxicam (11%). Although the oral formulations differed between the cited studies, or no details on the formulations were given, generally a high F% is reported for meloxicam in humans (89–97%)^24,25^ and in other mammals (85–106%)^26–28^. In contrast, the limited PK studies described in avian species, using the same oral formulation (Metacam oral suspension^®^, Boehringer Ingelheim) as used in this study, demonstrate a highly variable interspecies F%, ranging from 38% to 74%^15,18,29^. A possible explanation for these differences between avian species, could be the variability in pH values of their proventriculus^15,30^. In dogs, the F% of celecoxib and mavacoxib is influenced to a large extent by the feeding status of the animal. Presence of food was shown to increase oral bioavailability of both drugs^19,31^. Although in humans, no food effects on the F% of celecoxib were observed^31^. Since higher F% is seen in dogs with food, also in this study feed (2 mL intra-crop feed bolus by oral gavage) was co-administered. Further research is necessary to evaluate the impact of feed on F% of celecoxib, mavacoxib and meloxicam in avian species.

All three NSAIDs, evaluated in this study, are highly bound to plasma proteins (>95%). In general, extensive plasma protein binding typically leads to small Vd. The Vd of meloxicam after IV administration was indeed low (0.173 L/kg), which was consistent with the observed high plasma protein binding. In contrast, celecoxib (4.67 L/kg) and mavacoxib (10.99 L/kg) display a high Vd, despite being highly bound to plasma proteins. Similar effects were observed in dogs. Although extensively bound to plasma proteins (>98%), a Vd of 1.82–1.98 L/kg for celecoxib and 1.41–1.87 L/kg for mavacoxib were observed in dogs^19,31,32^. These observations may be related to the lipophilic nature of these drugs or the type of protein binding.

For mavacoxib and meloxicam, EHR has been described in literature^19,21,26,33,34^. The present study was not able to demonstrate a pronounced presence of EHR in cockatiels, possibly due to the limited blood collection points at the secondary peaks. However, the presence of EHR cannot be excluded. In accordance with dogs (Cl = 2.7 mL/h.kg)^19^, mavacoxib after IV administration in cockatiels is also characterized by a very low Cl (0.036 L/h.kg). Due to the combination of this low Cl with a relatively large Vd, mavacoxib has a remarkably long plasma T_1/2el_ exceeding 5 days. For mavacoxib, a prolonged therapeutic effect could be obtained requiring less frequent dosing as is the case in dogs (T_1/2el_ = 17.3 days; τ = 2 weeks between first two doses, thereafter 4 weeks). This is an interesting result, since for many drugs the correlation between dogs and birds is lacking. For example, cefovecin, a third generation cephalosporin, has a long elimination half-life in dogs (T_1/2el_ = 5.5 days)^35^ whereas the elimination half-life in hens is very short (T_1/2el_ = 0.9 h)^36^, suggesting a completely different posology. Although celecoxib and mavacoxib are strongly related structurally, celecoxib has a considerably higher plasma clearance (2.42 L/h.kg) which results in a relatively short T_1/2el_ of 1.34 h. For meloxicam, a higher value of Cl (0.388 L/h.kg) and corresponding shorter T_1/2el_ (0.31 h) after IV administration were obtained in cockatiels in comparison with other Psittaciformes (African grey parrots (Cl = 0.00218 L/h.kg; T_1/2el_ = 31.4 h) and Amazon parrots (Cl = 0.0122 L/h.kg; T_1/2el_ = 15.9 h))^15,18^. These observations highlight the significant differences in PK of meloxicam for avian species, as previously observed by Baert *et al*.^10^. Therefore, the PK needs to be evaluated in every target bird species to assure an appropriate selection of dosing regimen. Since PK, pharmacodynamics (PD) and drug safety profiles are often only available for a limited number of bird species, dose extrapolation between different avian species might be performed in veterinary practice based on the correlation of PK characteristics and BW. However, it is obvious that this should be done with caution^9,37^. In some cases, like meloxicam, previous research demonstrated that dose extrapolation is not a suitable method for dose determination in avian species^10^.

In avian species, establishing suitable dosing regimens is not straightforward. To date, there is insufficient knowledge about effective plasma concentrations of celecoxib, mavacoxib and meloxicam in bird species. For meloxicam, several PK studies have been conducted in birds, including chickens, ostriches, ducks, turkeys, pigeons, Hispaniolan Amazon Parrots and African grey parrots^10,15,18^. Information on the pharmacodynamics of meloxicam in birds is however relatively scarce. Cole *et al*. demonstrated that meloxicam administered at 1.0 mg/kg IM every 12 hours is significantly more effective than lower dosages in relieving arthritic pain in Hispaniolan parrots^38^. Hoppes *et al*. investigated the effect of meloxicam treatment in cockatiels infected with avian bornavirus. In contrast to the expectations, these authors have observed that the combination of avian bornavirus challenge and meloxicam (Metacam oral suspension^®^ (Boehringer Ingelheim), 0.5 mg/kg, orally, twice daily for 130 days) resulted in clinical disease and death, whereas animals solely receiving meloxicam or an ABV challenge alone remained clinically healthy until the end of the study. However, it is questionable whether effective plasma concentration at the used dose regimen (0.5 mg/kg; twice daily) were reached, since the presented study revealed a high clearance (0.388 L/h.kg) and remarkably low F% (11%) in cockatiels in comparison with other Psittaciformes. Furthermore, only a limited number (12 birds in total) of birds were included in this study. A large scale investigation is needed in order to evaluate the reproducibility of these results and, if necessary, to unravel the underlying mechanism of the observed difference^39^. For mavacoxib, plasma concentrations of 0.4 µg/mL were associated with efficacy in osteoarthritic dogs^20^. After single dose PO and IV mavacoxib administration (4 mg/kg BW) in cockatiels current study demonstrates that plasma concentrations of 0.4 µg/mL were always reached. However, a major issue is whether effective plasma concentrations are the same in different animal species and clinical conditions. There is a need for more PK-PD studies in order to determine the effective plasma concentration and dose regimen in avian species for each clinical condition. In addition, Cox *et al*. demonstrated that mavacoxib exhibited a dose proportional pharmacokinetics for single oral doses of 2–12 mg/kg in Beagle dogs^19^. In the presented study it was not possible to establish dose proportional PK for either mavacoxib, celecoxib as meloxicam. However this observation should be taken into account when determining effective dosing regimens.

As a conclusion it can be stated that sensitive and specific LC-MS/MS methods were developed for quantification of celecoxib, mavacoxib and meloxicam in avian plasma. Comparison of literature with presented PK parameters of meloxicam in cockatiels, such as F and Cl, highlight the presence of interspecies differences. Moreover, notable differences were observed in PK of mavacoxib compared to the structurally related celecoxib. As a consequence of lower mavacoxib clearance compared to celecoxib and meloxicam, a less frequent dosing might be suggested in cockatiels. However, pharmacodynamic and safety studies are necessary to further investigate the use and to establish appropriate dosage regimens of these NSAIDs in cockatiels.

## Materials and Methods

### Chemicals, products and reagents

The analytical standards of mavacoxib and celecoxib, as well as the internal standards (IS) mavacoxib-D_4_ and celecoxib-D_7_, were obtained from Clearsynth (Mumbai, India). Analytical standard of meloxicam was purchased from Sigma-Aldrich (Bornem, Belgium), whereas meloxicam-^13^C_6_ IS was obtained from Alsachim (Illkirch Graffenstaden, France). All analytical standards were stored at 2–8 °C. For the analytical experiments, stock solutions (SS) of mavacoxib and celecoxib (50 mg/mL) and their IS (5 mg/mL) were prepared in ethanol, whereas SS of meloxicam and meloxicam-^13^C_6_ (1 mg/mL) were prepared in methanol. Working solutions (WS) of the standards were prepared by appropriate dilution of the SS. All SS and WS were stored at ≤ −15 °C except for meloxicam and its IS which were stored at 2–8 °C. Water and acetonitrile (ACN) for analysis of mavacoxib, celecoxib and meloxicam were of HPLC-grade and were obtained from Filterservice (Eupen, Belgium). Ammonium acetate, formic acid (FA), ethanol, sodium hydroxide and ethyl acetate were of analytical grade and purchased from VWR (Leuven, Belgium). Ostro^TM^ protein precipitation and phospholipid removal 96-well plates were purchased from Waters (Zellik, Belgium). Microfilters Millex PVDF 0.22 μm were obtained to filter extracted samples (Millex, Overijse, Belgium).

For celecoxib and mavacoxib only CF for oral applications are available. In order to determine F%, standard solutions (STD) were made. STDs (5 mg/mL) were prepared by dissolving the celecoxib and mavacoxib analytical standard in a polyethylene glycol (PEG) 400:physiological saline (75:25, v/v) vehicle. This dissolution medium was used to overcome the pore water solubility of celecoxib and mavacoxib (logP ≥ 3)^21,31^. Commercially available tablet formulations of celecoxib (Celebrex^®^ 100 mg, Pfizer, Brussels, Belgium) and mavacoxib (Trocoxil^®^ 20 mg, Zoetis, Zaventem, Belgium) were ground and lactose was added to obtain a powder mixture with a concentration of 10 mg celecoxib/g and 4 mg mavacoxib/g. Immediately prior to oral administration, these powder mixtures were mixed with physiological saline (5 mg celecoxib/mL and 2 mg mavacoxib/mL). Since a CF for intravenous administration of meloxicam is available in Belgium, no STD for meloxicam was prepared. For IV and PO administration, respectively Metacam suspension for injection^®^ (5 mg/mL) and Metacam oral suspension^®^ (0.5 mg/mL) (Boehringer Ingelheim, Brussels, Belgium) were used.

### Animals and experimental procedure

#### General study design

A group of cockatiels consisting of 90 birds (45 ♂/45 ♀) of 6–12 months old were group-housed in an aviary during the two week acclimatization period at the start of the study, and during wash-out and recovery periods between different experiments. During these periods animals were fed with a commercially available seed mixture (Big Parakeets Prestige, Versele-Laga, Deinze, Belgium) and water *ad libitum*. Birds were gender-based randomly allocated to different experimental groups. Each bird participated in minimum one and maximum two out of the three PK studies. Birds were cage-housed in pairs, 8 h prior until 12 h post NSAID administration. In all trials, feed was withheld from the birds from 8 h prior until 4 h post NSAID administration. NSAIDs were administered IV (celecoxib and mavacoxib STD, meloxicam CF) in the *vena cutanea ulnaris superficialis* (wing vein) using a 1 mL syringe and 29 G needle, and PO (celecoxib and mavacoxib STD and CF, meloxicam CF) by intra-crop bolus using a 1 mL syringe and a curved stainless steel ball tipped feeding needle. Immediately following IV or PO drug administration, animals received a 2 mL intra-crop feed bolus (Nutribird A19 High Energy (Versele-Laga):tap water, 25:75, v-v) by oral gavage. Subsequently, blood (0.3 mL) was sampled by venipuncture from the jugular vein (*vena jugularis*) using a 1 mL syringe and 29 G needle, and transferred into heparinized tubes, before (0 h) and at different time points after drug administration. A sparse sampling protocol was applied because of the limited volume of blood that can be drawn from these birds. Therefore, all sampling points were randomly allocated to different birds, with maximum two (celecoxib and meloxicam) or three (mavacoxib) sampling points per bird. After blood sampling, all samples were centrifuged (2850 × *g*, 10 min, 4 °C) within 2 h and aliquots of plasma were stored at ≤ −15 °C until analysis.

#### Celecoxib PK study

The PK characteristics of celecoxib (10 mg/kg BW) were first investigated after a single PO administration of celecoxib CF to 22 cockatiels (11♂/11♀, 93 ± 10 g). Besides, celecoxib STD was administered by single PO and IV administration in a two-way cross-over design to 34 other cockatiels (17♂/17♀, 91 ± 10 g). For the latter, each bird received a single PO bolus of celecoxib STD and an IV bolus of celecoxib STD respecting one month of wash-out and recovery period between the two routes of administration. Blood was withdrawn before administration (t = 0) and at 15, 30, 45 min, and 1, 2, 4, 6, 8 and 12 h after celecoxib administration. Additional sampling was performed 5 min after IV and 24 h after PO administration.

#### Mavacoxib PK study

After a wash-out and recovery period of one month, the second experiment was conducted to study the PK characteristics of mavacoxib (4 mg/kg BW). A similar setup as for the celecoxib study was used. Again a single PO bolus of mavacoxib CF was administered to 26 cockatiels (13♂/13♀, 93 ± 9 g). Next IV and PO dosing of celecoxib STD was performed in two-way cross-over design to 40 cockatiels (20♂/20♀, 101 ± 8 g) with a period of three months wash–out and recovery period between PO and IV STD administrations. Blood was withdrawn before administration (t = 0) and at 15, 30, 45 min, and 1, 2, 4, 6, 8, 12, 24, 48, 72, 96, 120, 168, 336, 672 and 1008 h after administration. An additional sample was taken 5 min after IV administration.

#### Meloxicam PK study

After a period of six months, the third experiment investigated the PK characteristics of meloxicam (1 mg/kg BW) CF in a two-way cross over design in 24 cockatiels (12♂/12♀, weighing 101 ± 12 g), with a period of one month between both administrations. Blood was withdrawn before administration (t = 0) and at 15, 30, 45 min, and 1, 2, 4, 6, 8 and 12 h following administration. Additional samples were taken 5 min after IV and 24 h after PO administration.

#### Ethics statement

Animal care and all experiments were conducted in accordance with the Belgian Royal Decree of 29 may 2013 and the EU directive of 2010/63/EU. The animal experiments were approved by the Ethical Committee of the Faculty of Veterinary Medicine and the Faculty of Bioscience Engineering of Ghent University (EC 2014/113 and EC 2015/114).

### LC-MS/MS analysis of NSAIDs in plasma

Quantification of celecoxib, meloxicam and mavacoxib in plasma of cockatiels was carried out using an in-house developed and validated high-performance liquid chromatography-tandem mass spectrometry (HPLC-MS/MS) method.

#### Sample preparation

For cele- and mavacoxib, sample preparation consisted of sequential addition of 25 µL of IS WS (1000 ng/mL celecoxib-D_7_, 200 ng/mL mavacoxib-D_4_), 25 µL of ethanol, 150 µL of 0.1 M NaOH and 3 mL of ethyl acetate to 50 μL of plasma. After vortex mixing (15 s), the samples were extracted for 20 min on a horizontal roller mixer and centrifuged for 10 min (3724 × g, 4 °C). The supernatant was evaporated under a gentle nitrogen stream (40 ± 5 °C). The dry residue was reconstituted in 250 μL of water/ACN (50:50, v/v). After a vortex-mixing step (15 s), the samples were filtered through a Millex^®^ PVDF syringe filter (0.22 µm) and transferred into an autosampler vial. A 10 μL aliquot of the final clear solution was injected onto the HPLC-MS/MS instrument.

Sample preparation for meloxicam consisted of the addition of 50 µL of HPLC methanol and 25 µL of IS WS (10 µg/mL) ^13^C_6_-meloxicam to 50 μL of plasma. After vortex mixing, the plasma samples were transferred to an Ostro^TM^ 96-well plate, followed by the addition of 375 µL of 1% FA in ACN to each well. After aspirating the samples 5 times using a multichannel pipette, the sample was passed through the well plate by application of a vacuum for 5–10 min. A 10 μL aliquot was injected onto the HPLC-MS/MS instrument.

#### LC-MS/MS instrumentation

Chromatographic separation was achieved on a Zorbax Eclipse plus C18 column (3.0 mm internal diameter × 100 mm, particle diameter, 3.5 μm) in combination with a guard column of the same type, both from Agilent Technologies (Sint-Katelijne-Waver, Belgium). The mobile phases for cele- and mavacoxib analysis consisted of 10 mM ammonium acetate in HPLC grade water (mobile phase A) and HPLC grade ACN (mobile phase B). Following gradient elution program was run: 0.0–1.0 min (50% A, 50% B), 1.0–6.0 min (linear gradient to 90% B), 6.0–8.5 min (10% A, 90% B), 8.5–9.0 min (linear gradient to 50% A), and 9.0–12.0 min (50% A, 50% B). Flow rate was set at 300 μL/min. For meloxicam, HPLC grade water containing 0.01% formic acid (mobile phase A) and HPLC grade ACN containing 0.01% formic acid (mobile phase B) were used. Following gradient elution program was run: 0.0–1.5 min (60% A, 40% B), 1.5–5.20 min (linear gradient to 5% A), 5.2–7.0 min (5% A, 95% B), 7.0–7.1 min (linear gradient to 40% B) and 7.1–10 min (60% A, 40% B). Flow rate was set at 300 µL/min.

Detection was performed on a TSQ Quantum Ultra triple-quadrupole mass spectrometer (ThermoFisher Scientific, Breda, the Netherlands), equipped with a heated electrospray ionization (h-ESI) probe operating in the negative ionization mode for celecoxib and mavacoxib, and in the positive ionization mode for meloxicam. Following transitions (m/z) were used for identification and quantification, respectively, for celecoxib: 380.1 → 316.1 and 380.1 → 276.0, for celecoxib-D_7_: 387.1 → 323.1 and 387.1 → 283.0, for mavacoxib: 384.1 → 320.0 and 384.1 → 279.9, for mavacoxib-D_4_: 388.1 → 283.1 and 388.1 → 324.1, for meloxicam: 352.0 → 115.0 and 352.0 → 141.0, and for meloxicam-^13^C_6_: 358.0 → 115.0 and 358.0 → 141.0.

The methods were validated in house according to recommendations reported in literature and guidelines of the European Community^40–43^. Based on the limited blood volume of cockatiels, LC-MS/MS calibration and validation were performed using broiler chicken plasma. Following parameters were evaluated: linearity (r and gof), within-run and between-run precision and accuracy, LOQ, LOD and carry-over. The validation protocol and the acceptance criteria used were previously described by De Baere *et al*.^44^.

### Plasma protein binding

Freshly blank cockatiel plasma was spiked with celecoxib, mavacoxib or meloxicam STD at 0.50 µg/ mL and 5.0 µg/mL. One aliquot of each concentration was analysed in the same way as the PK study samples (aliquot 1) to determine the total plasma concentration. Two other aliquots of each concentration (aliquot 2 and 3) were incubated for 1 hour in a hot water bath of 41 °C, and subsequently transferred onto Microcon^®^ centrifugal filters (type Ultracel YM-30, Merck, Overijse, Belgium) and centrifuged (2980 × g, 10 min, 41 °C). Thereafter, the obtained filtrate of aliquot 2 and 3 were analysed in the same way as the PK study samples to determine the free plasma concentration. For quantification, corresponding calibration curves for each aliquot were used. Equation (1) was used to calculate the plasma protein binding.

1 $${Plasma}\,{protein}\,{binding}\,( \% )=\frac{({total}\,{plasma}\,{conc}.-{free}\,{plasma}\,{conc}.)}{{total}\,{plasma}\,{conc}.}\times 100$$

The results are presented as mean ± standard deviation.

### Pharmacokinetic analysis

#### Population pharmacokinetics celecoxib

Plasma concentration–time data were analysed by nonlinear mixed-effects regression using first-order conditional estimation with extended least squares (FOCE-ELS) as an estimation method in Phoenix NLME^®^ (Certara, Cary, NC, USA). Values below the LOQ were excluded from the data set prior to data summarization and pharmacokinetic analysis. The structural PK model for the IV data was a one-compartment model with first order elimination as expressed by equation (2).

2 $$C(t)={C}_{0}.{e}^{-\frac{Cl}{Vd}t}$$

Where C is the plasma concentration, t is the time, Cl is total body clearance, Vd is the volume of distribution and C_0_ is the concentration at time zero. The structural model for the PO data (celecoxib STD and CF) was a one-compartmental with first order absorption and elimination as expressed by equation (3). A lag time for absorption (Tlag) was considered for each PO model.

3 $$C(t)=\frac{F\,D\,{k}_{a}}{Vd\,({k}_{a}-{k}_{e})}\cdot ({e}^{-{k}_{e}t}-{e}^{-{k}_{a}t})$$

Where F denotes the bioavailability, k_a_ is the first-order absorption rate constant, k_e_ is the first-order elimination rate constant and D is the dose. Interindividual variability was expressed using an exponential error model according to equation (4).

4 $${{\rm{P}}}_{i}={{\rm{\theta }}}_{{\rm{P}}}\cdot {{{\rm{e}}}^{{\rm{\eta }}}}_{{\rm{P}}i}$$

where P_i_ is the primary pharmacokinetic parameter value in the i^th^ patient, θ_P_ is the typical value of the parameter in the population, and η_Pi_ is a random variable in the i^th^ patient with a mean of zero and a variance of ω². Interindividual variability is reported as ω. Residual variability (ε), with a mean of zero and a variance of σ², was evaluated within the best structural PK model. For the IV and PO STD dosing, a multiplicative (equation (5)) and additive (equation (6)) error model was used to describe the residual variability, respectively, whereas for the PO CF administration, the best residual error model was log-additive (equation (7)).

5 $${\rm{Multiplicative}}:{C}_{obs}={C}_{pred}\cdot (1+\varepsilon )\,$$

6 $${\rm{Additive}}:{C}_{obs}={C}_{pred}+\varepsilon \,$$

7 $$\mathrm{Log} \mbox{-} {\rm{additive}}:{C}_{obs}={C}_{pred}+\varepsilon \cdot sqrt(1+{C}_{pred}^{\,2\,\cdot \,({\sigma }_{mult}/{{\sigma }_{add)}}^{2}})$$

Where σ_mult_ is the variance of the multiplicative residual error; σ_add_ is the variance of the additive residual error; C_obs_ is the observed concentration for the individual; C_pred_ is the model predicted concentration and ε is the error value.

Structural and error model selection was guided by visual inspection of goodness-of-fit plots (e.g., observed vs. predicted plasma concentrations, weighted residuals versus predicted concentrations, and weighted residuals versus time), −2LL, AIC and BIC as well as precision of the parameter estimates. The models were chosen based on the smaller values of −2LL, AIC and BIC, better precision of estimates, and superior goodness-of-fit plots.

Evaluated covariates were BW (continuous variable) and gender (categorical variable). A stepwise forward-backward process was used to evaluate whether inclusion of the covariates significantly improved the model fit using a −2LL test. A decrease in −2LL with a p-value < 0.01 was considered significant for addition and p < 0.001 for exclusion of the covariate.

Following fixed effect parameters were determined: Vd: volume of distribution (IV); Vd/F: volume of distribution uncorrected for absolute oral bioavailability (PO); Cl: total body clearance (IV); Cl/F: total body clearance uncorrected for absolute oral bioavailability (PO); K_a_: absorption rate constant and Tlag: lag time for absorption following oral administration. Computed secondary parameters were: C_0_: plasma concentration at time 0 following IV administration; AUC_0-inf_: area under the plasma concentration-time curve from time 0 to infinity; K_e_: elimination rate constant; T_1/2el_: elimination half-life; T_1/2a_: absorption half-life; C_max_: maximal plasma concentration; T_max_: time to maximal plasma concentration; K_1g_: bile excretion rate and τ: bile excretion interval.

The absolute oral bioavailability (F, expressed as %, F%) was calculated according to equation (8).

8 $${\rm{F}} \% ={{\rm{AUC}}}_{0 \mbox{-} \inf {\rm{PO}}}/{{\rm{AUC}}}_{0 \mbox{-} \inf {\rm{IV}}}\times 100$$

Where AUC_0-inf_ is area under the plasma concentration-time curve from time 0 to infinity.

#### Population pharmacokinetics mavacoxib

PopPK analysis for mavacoxib was performed in a similar manner as for celecoxib. The structural PK model for the IV (STD) and PO (STD/CF) data was a one-compartment model with first order elimination and a one-compartment with first order absorption and elimination, respectively. Inter- and intra-individual variability were expressed according to the exponential error model and the multiplicative residual error model, respectively. Selection and validation of the models and covariate evaluation was performed as described above.

#### Population pharmacokinetics meloxicam

Visual inspection of the plasma concentration-time profiles revealed a secondary rise in the average plasma concentration during the elimination phase (around 1-2 and 4 h following IV and PO administration, respectively) following both IV and PO administration of meloxicam. This could be attributable to EHR, which has previously been described for meloxicam^33^. Therefore, for the IV data a one-compartment structural model with first order elimination was compared with or without the inclusion of EHR. This model was derived from Gabrielsson and Weiner^45^. Also for PO administration, a one-compartment model with a lag time, first order absorption and first order elimination was compared with and without EHR inclusion. Inter- and intra-individual variability were expressed according to the exponential error model and the multiplicative residual error model, respectively. Comparison and selection of the models and covariate evaluation was performed as described above.

### Data Availability

The datasets generated and/or analysed during the current study are available from the corresponding author on reasonable request.

## Electronic supplementary material

Supplementary information
