# Supplementary material for: Comparative population pharmacokinetics and absolute oral bioavailability of COX-2 selective inhibitors celecoxib, mavacoxib and meloxicam in cockatiels (Nymphicus hollandicus)
Source: Sci Rep. 2017 Sep 25;7:12043. doi: 10.1038/s41598-017-12159-z (PMC5612971; doi:10.1038/s41598-017-12159-z)

Comparative population pharmacokinetics and absolute oral bioavailability of COX-2 selective inhibitors celecoxib, mavacoxib and meloxicam in cockatiels *(Nymphicus hollandicus)*

Laura Dhondt [1], Mathias Devreese [1,2], Siska Croubels [1], Siegrid De Baere [1], Roel Haesendonck [3], Tess Goessens [1], Ronette Gehring [2], Patrick De Backer [1], Gunther Antonissen [1,3] *

[1] Department of Pharmacology, Toxicology and Biochemistry, Faculty of Veterinary Medicine, Ghent University, Merelbeke, Belgium

[2] Department of Anatomy and Physiology, Institute of Computational Comparative Medicine, College of Veterinary Medicine, Kansas State University, Manhattan, KS, USA

[3] Department of Pathology, Bacteriology and Avian Diseases, Faculty of Veterinary Medicine, Ghent University, Merelbeke, Belgium

(*) Corresponding author: Gunther.Antonissen@UGent.be

Supplementary Table S1. Results of the evaluation of linearity (goodness-of-fit coefficient (gof), correlation coefficient (r)), limit of quantification (LOQ) and limit of detection (LOD) of celecoxib, mavacoxib and meloxicam in broiler chicken plasma

Analyte	Calibration range (ng mL^{-1})	Spike levels (ng mL^{-1})	gof (%)	r	LOQ (ng mL^{-1})	LOD (ng mL^{-1})
Celecoxib	5 - 5000	5, 10, 20, 100, 200, 500, 1000, 2000, 5000	4.95	0.9982	5	0.22
Mavacoxib	5 - 5000	5, 10, 20, 100, 200, 500, 1000, 2000, 5000	5.08	0.9999	5	0.25
Meloxicam	10 - 5000	10, 25, 50, 100, 250, 500, 1000, 2500, 5000	6.25	0.9975	10	0.18

Acceptance criteria: r > 0.99 and g < 20 %

Supplementary Table S2. Results of the within-run and between-run precision and accuracy evaluation for the analysis of celecoxib, mavacoxib and meloxicam in broiler chicken plasma

Analyte	Theoretical concentration (ng/mL)	Mean concentration ± SD (ng/mL)	Precision, RSD (%)	Accuracy (%)
Celecoxib [a]	5 (n = 6)	4.8 ± 0.591	12.4	-4.9
	200 (n = 6)	190.0 ± 9.99	5.3	-5.0
	2000 (n = 6)	1966.0 ± 89.79	4.6	-1.7
Celecoxib[b]	5 (n = 6)	5.5 ± 0.159	2.9	9.8
	200 (n = 6)	202.4 ± 19.30	9.5	1.2
	2000 (n = 6)	2049.6 ± 146.05	7.1	2.5
Mavacoxib [a]	5 (n = 6)	4.9 ± 0.775	15.7	-1.0
	200 (n = 6)	199.0 ± 15.69	7.9	-0.5
	2000 (n = 6)	1909.5 ± 19.56	1.0	-4.5
Mavacoxib[b]	5 (n = 6)	5.3 ± 0.245	4.6	6.0
	200 (n = 6)	201.0 ± 7.05	3.5	0.5
	2000 (n = 6)	1973.2 ± 77.64	3.9	-1.3
Meloxicam [a]	25 (n = 6)	21.5 ± 0.77	3.6	-13.9
	250 (n = 6)	246.9 ± 5.43	12.2	-1.3
	2500 (n = 6)	2532.4 ± 68.21	2.7	1.3
Meloxicam[b]	25 (n = 14)	25.9 ± 2.95	11.4	3.6
	250 (n = 14)	249.6 ± 10.09	4.0	-0.2
	2500 (n = 14)	2457.9 ± 128.98	5.2	-1.7

[a] Within-run accuracy and precision; [b] Between-run accuracy and precision; SD: standard deviation; RSD: relative standard deviation; Acceptance criteria: accuracy: 1-10 ng/mL: -30% to +10%, > 10 ng/mL: -20% to +10%, within-run precision (RSD_{max}): < 10 ng/mL: 25.0%, ≥ 10 ng/mL: 15.0%, ≥ 100 ng/mL: 10.0%, between-run precision: 5 ng/mL: 35.5%, 25 ng/mL: 27.9%, 200 ng/mL: 20.4%, 250 ng/mL: 19.7%, 2000 ng/mL: 14.4% and 2500 ng/mL: 13.9%

Supplementary Figure S1. Visual evaluation of the population model of celecoxib after intravenous (IV [1]: analytical standard) and oral (PO [1]: analytical standard; [2]: commercial formulation) dosing: scatter plot of the population dependent variable (DV), namely observed plasma concentration (Cobs) versus the individually predicted plasma concentration values (IPRED) (a) and QQ plot of the conditionally weighted residuals of Cobs (b)

IV[1]

(a)

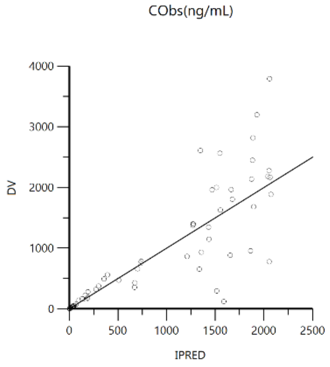

(b)

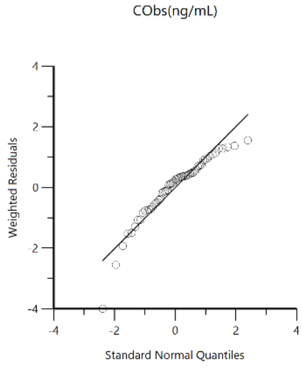

PO[1]

(a)

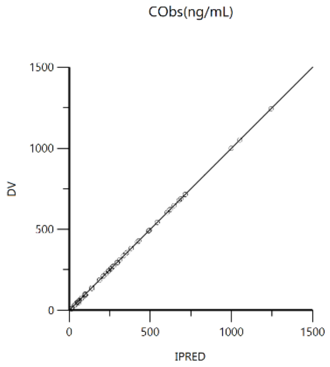

(b)

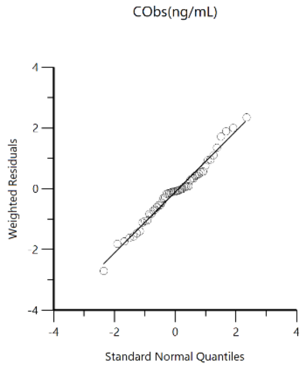

PO[2]

(a)

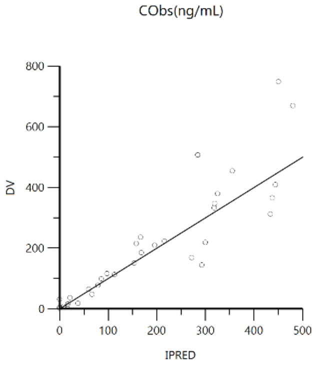

(b)

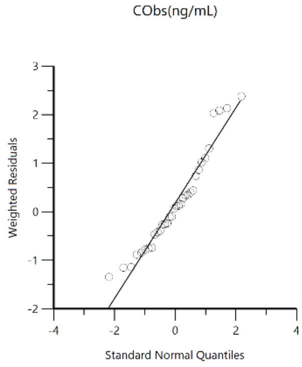

Supplementary Figure S2. Visual evaluation of the population model of mavacoxib after intravenous (IV [1]: analytical standard) and oral (PO [1]: analytical standard; [2]: commercial formulation) dosing: scatter plot of the population dependent variable (DV), namely observed plasma concentration (Cobs) versus the individually predicted plasma concentration values (IPRED) (a) and QQ plot of the conditionally weighted residuals of Cobs (b)

IV[1]

(a)

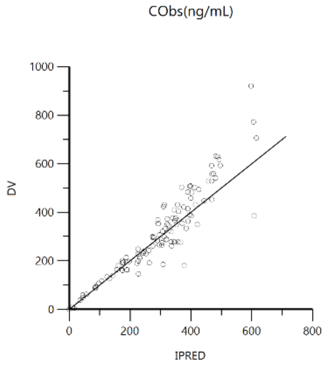

(b)

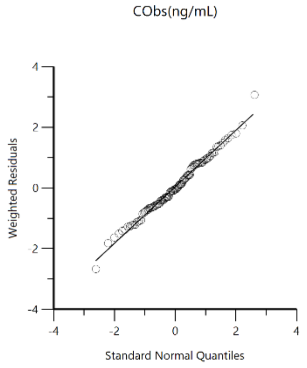

PO[1]

(a)

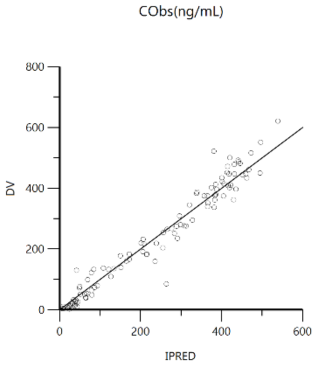

(b)

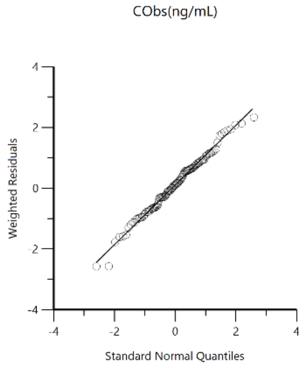

PO[2]

(a)

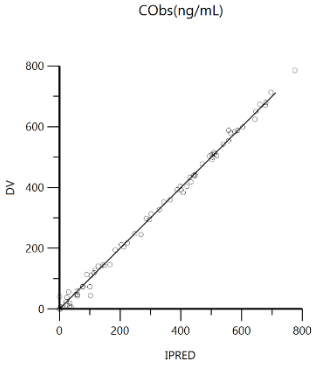

(b)

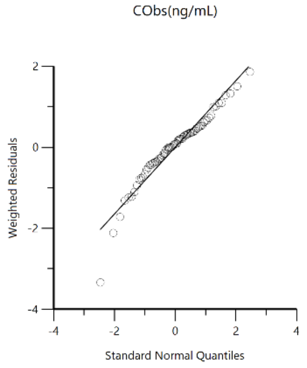

Supplementary Figure S3. Visual evaluation of the population model of meloxicam after intravenous (IV) and oral (PO) dosing: scatter plot of the population dependent variable (DV), namely observed plasma concentration (Cobs) versus the individually predicted plasma concentration values (IPRED) (a) and QQ plot of the conditionally weighted residuals of Cobs (b)

IV, no enterohepatic recycling

(a)

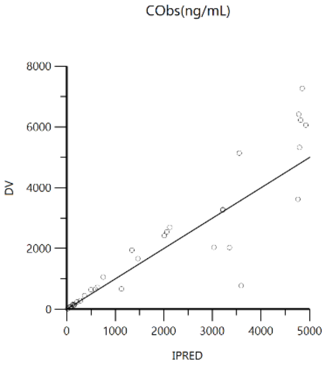

(b)

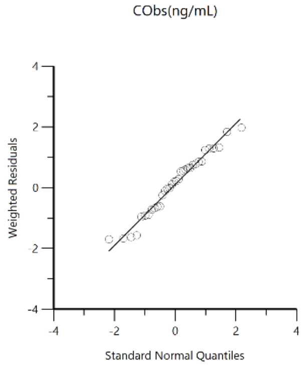

IV, with enterohepatic recycling

(a)

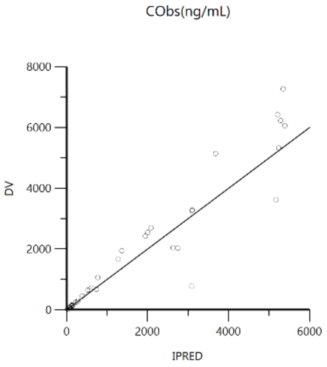

(b)

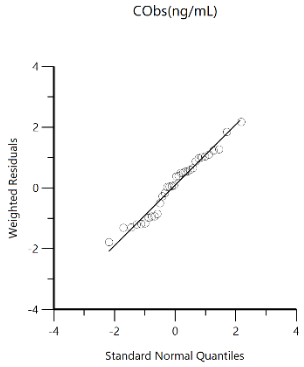

PO

(a)

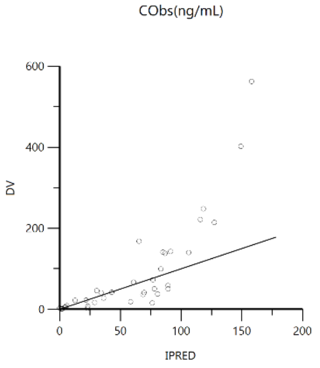

(b)

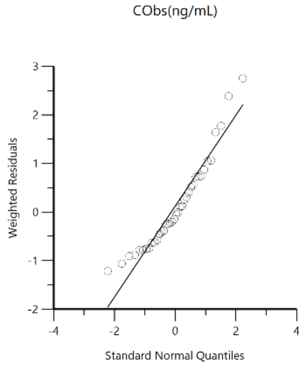

Supplement: Supplementary file 1 — Supplementary information [file 41598_2017_12159_MOESM1_ESM.pdf]
